# Harmonising electronic health records for reproducible research: challenges, solutions and recommendations from a UK-wide COVID-19 research collaboration

**DOI:** 10.1186/s12911-022-02093-0

**Published:** 2023-01-16

**Authors:** Hoda Abbasizanjani, Fatemeh Torabi, Stuart Bedston, Thomas Bolton, Gareth Davies, Spiros Denaxas, Rowena Griffiths, Laura Herbert, Sam Hollings, Spencer Keene, Kamlesh Khunti, Emily Lowthian, Jane Lyons, Mehrdad A. Mizani, John Nolan, Cathie Sudlow, Venexia Walker, William Whiteley, Angela Wood, Ashley Akbari, CVD-COVID-UK/COVID-IMPACT Consortium

**Affiliations:** 1 Population Data Science, Swansea University Medical School, Faculty of Medicine, Health and Life Science, Swansea University, Swansea, UK; 2 British Heart Foundation Data Science Centre, Health Data Research UK, London, UK; 3 Institute of Health Informatics, University College London, London, UK; 4 NHS Digital, Leeds, UK; 5 British Heart Foundation Cardiovascular Epidemiology Unit, Department of Public Health and Primary Care, University of Cambridge, Cambridge, UK; 6 Diabetes Research Centre, University of Leicester, Leicester, UK; 7 Department of Population Health Sciences, Bristol Medical School, University of Bristol, Bristol, UK; 8 Centre for Clinical Brain Sciences, University of Edinburgh, Edinburgh, UK

**Keywords:** Population health, Data harmonisation, Common data model, Electronic health record, Trusted Research Environments, Reproducible research, SAIL databank, NHS digital TRE for England, COVID-19

## Abstract

**Background:**

The CVD-COVID-UK consortium was formed to understand the relationship between COVID-19 and cardiovascular diseases through analyses of harmonised electronic health records (EHRs) across the four UK nations. Beyond COVID-19, data harmonisation and common approaches enable analysis within and across independent Trusted Research Environments. Here we describe the reproducible harmonisation method developed using large-scale EHRs in Wales to accommodate the fast and efficient implementation of cross-nation analysis in England and Wales as part of the CVD-COVID-UK programme. We characterise current challenges and share lessons learnt.

**Methods:**

Serving the scope and scalability of multiple study protocols, we used linked, anonymised individual-level EHR, demographic and administrative data held within the SAIL Databank for the population of Wales. The harmonisation method was implemented as a four-layer reproducible process, starting from raw data in the first layer. Then each of the layers two to four is framed by, but not limited to, the characterised challenges and lessons learnt. We achieved curated data as part of our second layer, followed by extracting phenotyped data in the third layer. We captured any project-specific requirements in the fourth layer.

**Results:**

Using the implemented four-layer harmonisation method, we retrieved approximately 100 health-related variables for the 3.2 million individuals in Wales, which are harmonised with corresponding variables for > 56 million individuals in England. We processed 13 data sources into the first layer of our harmonisation method: five of these are updated daily or weekly, and the rest at various frequencies providing sufficient data flow updates for frequent capturing of up-to-date demographic, administrative and clinical information.

**Conclusions:**

We implemented an efficient, transparent, scalable, and reproducible harmonisation method that enables multi-nation collaborative research. With a current focus on COVID-19 and its relationship with cardiovascular outcomes, the harmonised data has supported a wide range of research activities across the UK.

**Supplementary Information:**

The online version contains supplementary material available at 10.1186/s12911-022-02093-0

## Background

The COVID-19 pandemic emphasised the importance of efficient, accurate, and collaborative approaches in research [1]. Electronic health records (EHRs) from a range of sources have been used in many COVID-19 studies throughout the pandemic. There are still existing gaps in these studies, such as: (i) cross-validation of findings in other healthcare systems and nations to determine generalisability (ii) using a large sample size for capturing rare events to gain sufficient statistical power (iii) examining the inequalities across vulnerable subgroups to estimate and address health inequality and ensure health justice. Hence it is vital to establish common approaches for efficient collaborative research at a national or global scale using EHRs from different databases across different nations, facilitating comparisons of the impact of COVID-19, and supporting public health decision-making [2–5].

The challenge of establishing common approaches becomes even greater when analysing sensitive data within and across Trusted Research Environments (TREs, secure platforms used to store or analyse sensitive data) [6, 7]. TREs, data access services, and platforms may differ in data access, sharing policies, and governance, creating a challenge for projects wishing to combine data at the individual-level. Hence, the adoption of common approaches at the design level of projects is of high importance [8]. One solution to this challenge is federated analytics, whereby the data from multiple TREs or platforms remains at its source and is harmonised to a Common Data Model (CDM) that allows results from multiple locations to be generated and combined using meta-analysis techniques [9–11]. This harmonisation can occur via a rules-based approach with common, agreed analytic protocols applied within the separate TREs. CDM harmonisation includes the harmonisation of phenotypes, typically composed of several clinical codes to define an event, such as a diagnosis or prescription to a standard list, including any harmonisation between different coding systems (e.g., Read V2 and SNOMED in primary care data in the UK [12, 13]), all the way to a full harmonisation to a common standard such as Observational Medical Outcomes Partnership (OMOP) CDM [14].

Harmonisation is the process of making data and statistics more comparable, consistent and coherent [15]. In population studies using routinely collected data, harmonisation can only take a retrospective approach, i.e. post-data collection [16, 17]. Retrospective harmonisation of EHRs poses several technical challenges as healthcare data generally differ by underlying healthcare systems, type of information collected, drug/vaccine and medical event coding systems and language. Furthermore, different data sources have different data structures, fields, validation procedures, and accuracy issues [9, 16]. In the context of the COVID-19 pandemic, the advantages of harmonising EHRs across different nations have been shown for investigating the risk of cerebral venous sinus thrombosis after COVID-19 vaccines across three UK nations [18], creation of a pan-European cohort to advance the knowledge of the effects and treatment of COVID-19 [19], addressing some clinical and epidemiological questions around COVID-19 using hospital data from 96 hospitals across five countries [2], and study of COVID-19 associated clinical outcomes in the paediatric population [20]. The value of harmonisation goes beyond the context of COVID-19, while the required considerations for the use of National Health Service (NHS) data in the UK for research and analysis has been detailed in [9] as well as other guidelines for retrospective data harmonisation [16, 21], all aiming to ensure quality, reproducibility, and transparency of the harmonisation process. There are examples of using the CDM approach and harmonising EHRs from different nations (that go beyond the COVID-19 challenges) [22–29], proving the usefulness and generalisability of such approaches in research.

Motivated by the public health importance of understanding the relationship between COVID-19 and cardiovascular diseases (CVD), the Health Data Research UK (HDR UK) British Heart Foundation (BHF) Data Science Centre (DSC) established the CVD-COVID-UK consortium and related research programme [1]. Through the CVD-COVID-UK consortium, anonymised individual-level data from UK nations (England, Scotland and Wales) have been accessed on > 65 million individuals [30, 31], and further work is ongoing to enable access to Northern Ireland data. Accredited researchers working on approved projects can access routinely collected EHR and administrative data sources within secure, privacy-protecting TREs provided by NHS Digital in England [32], the National Data Safe Haven in Scotland [33] and the Secure Anonymised Information Linkage (SAIL) Databank in Wales [34]. The main linkable data sources in these TREs include primary and secondary care data, critical and intensive care data, prescribing and dispensing records, COVID-19 testing and vaccination data, mortality records, maternity services and a range of other data sources (see [35] for a full list of available data sources in each TRE available via the consortium).

In this paper, we characterise the challenges of harmonising anonymised individual-level EHRs from multiple TREs, focusing on the SAIL Databank for Wales and the NHS Digital TRE for England. We also describe how we addressed these challenges by creating a reproducible method for harmonising data from Wales (held within the SAIL Databank) with data from England (held within the NHS Digital TRE), as part of the CVD-COVID-UK. We conclude with recommendations and best practices for reducing the burden of retrospective harmonisation of EHRs based on our experiences, which may be employed and serve as useful starting points for future collaborations.

## Challenges for data harmonisation between TREs

We identified five broad challenges in establishing data harmonisation between TREs: how to achieve consistent definition of analysis variables, a reliable population denominator, transparency and communication of approaches, IT infrastructure, and disclosure control and pooling analyses. We recognise many previous research projects will have potentially undertaken some of these challenges, often in isolation. However, we need to start making available our insights in relation to these challenges to improve the efficiency, reproducibility, and transferability of the overall research process and improve the useability and efficiency of research across the data science and research community.

### Consistent definition/derivation of analysis variables

One of the fundamental challenges for research carried out using multiple data sources across multiple TREs is achieving consistency in the way variables are derived for statistical analysis. A good first step towards this is establishing how to extract meaningful values from the range of available data sources. To record diagnosis of diseases and health problems, symptoms and observations, prescribed or dispensed medication, and performed procedures, separate healthcare systems and data sources will use different coding systems and clinical terminologies, resulting in many permutations and options for the code-lists that are needed for research using data sources within a single TRE and more strongly across multiple TREs with more diverse data. Additional challenges would arise if a coding system is retired or different versions of a coding system have been used historically. To achieve meaningful value extraction, phenotypes need to be established in a unified manner, allowing processes for expert review and validation of the mapping between the different coding systems in use [36–38]. This is often done through the creation of dynamic phenotype libraries, an indexed and flexible library of computable phenotypes (a definition of a condition, disease, or characteristic or clinical event based solely on data that can be processed by a computer [38]), containing metadata, supporting information and the lifecycle of phenotypes (version number or date of last change, and whether the phenotype is retired due to changes in clinical practice, the underlying clinical definitions, or the coding systems). An example is the HDR UK Phenotype Library [12], an open platform for the creation, storage, dissemination, reuse, evaluation, and citation of curated algorithms and metadata.

Laboratory results available in EHRs may vary in reporting units, terminologies, calculated parameters, and report formatting due to heterogeneous data collections. Additionally, some data sources containing laboratory results do not have an associated data dictionary. These factors pose greater challenges for the consistent derivation of analysis variables from laboratory results data (within a TRE or across TREs). Working with healthcare professionals and domain experts is required to create unified phenotypes and related code-lists for laboratory data, as well as careful considerations when choosing a canonical unit for each measurement and identifying acceptable value ranges [39, 40].

Unified phenotypes are used to extract values into harmonised variables ready for analysis. In the extraction and transformation of these values, two main things need to be considered: the timing of the recording and the level of detail recorded. It is only natural for researchers within each TRE to want to maximise the utility of the available data, which translates to being as longitudinal, up-to-date, and granular as possible. However, with EHRs, coding the most recent clinical events is often incomplete and will be improved retrospectively at varying rates. Additionally, the granularity of clinical event recordings is unlikely to be consistent across data sources in participating TREs. Therefore, continued effort is needed to ensure researchers within and across TREs are aware of these challenges and are informed about the limitations and opportunities when using these types of data.

### Reliable population denominator

A reliable population denominator with a consistent set of demographic characteristics is essential for any epidemiological study, especially when the inferences are at population-level [41]. Trying to achieve this with EHRs alone can be challenging as, there are TREs that hold data sources with linkable demographic details for the general population directly published by official bodies such as the Office of National Statistics (ONS) census; while, in others the population denominator is defined based on those who have a recorded interaction with the healthcare services or registered with primary care. Both of these can further be complicated by the longitudinal nature of health records, leading to an accumulation of individuals exceeding the general population in number. If available, it is indeed more reliable to use concordance across multiple data sources, including birth and death records and registration with primary care services, to confirm whether someone is living amongst the general population and for what time periods. Also, this is a good opportunity to resolve conflicts around differences in multiple recordings of, say, date or week of birth, sex, and ethnic group. Once established, the population denominator can form the population spine for almost all types of study and should be updated on a regular basis to include changes to the population and the respective available denominator of individuals, including migration in or out and mortality [42].

### Transparency and communication

Within a given project, any individual researcher does not typically have access to more than one TRE. Thus, validating approaches within each TRE requires clear communication and transparent documentation. Establishing effective communication across various members of the project as well as stakeholders can be challenging, but it is essential. Creating a single point of truth is critical, as is visualising any data flows. Understanding how approaches within each TRE map on to each other is a more realistic aim than ensuring they are identical, and in turn follow the same best practices or naming conventions.

### IT infrastructure

Four key aspects to consider regarding the IT infrastructure within each collaborating TRE are: version control system, data storage platform, statistical analysis software, and the availability of performant hardware. Given transparency and communication challenges, having a version control system in place to track changes in the developing code is critical. Any differences among the other aspects mean that divergences in how data preparation and analysis are implemented should be expected, and in fact, greater levels of programming expertise may be required.

### Disclosure control and pooling analyses

To combine analysis results from each TRE, researchers need to be aware of the disclosure control processes each TRE has in place, and how they differ. The main principle behind each disclosure process is to ensure that any output requested out of the secure TRE environment does not contain information that could be used either on its own or in conjunction with other data to identify a person. However, there will be fundamental differences in the restrictions over content, format, structure and granularity of the results. For example, when using any data obtained through the Digital Economy Act (DEA), which includes the ONS 2011 Census, no small numbers between zero and 10 are allowed to be released. Independent TREs may also have different restrictions on whether aggregate counts with a value of 0 may be released. Whether categories are aggregated before release to escape small counts or imputed post-release is a decision for the project.

## Methods

To overcome the challenges characterised in the previous section for harmonising EHRs from the SAIL Databank for Wales and the NHS Digital TRE for England, we adopted a four-layer process for the CVD-COVID-UK projects within SAIL, aiming to optimise reusability and reproducibility. We used multiple demographic and EHR data sources, including primary and secondary care-related data sources, prescribing and dispensing records, COVID-19 testing and vaccination data, and mortality records. The data sources contributed varied follow-up time, which covered the years 1990–2022 (Additional file 1).

**Fig. 1:**
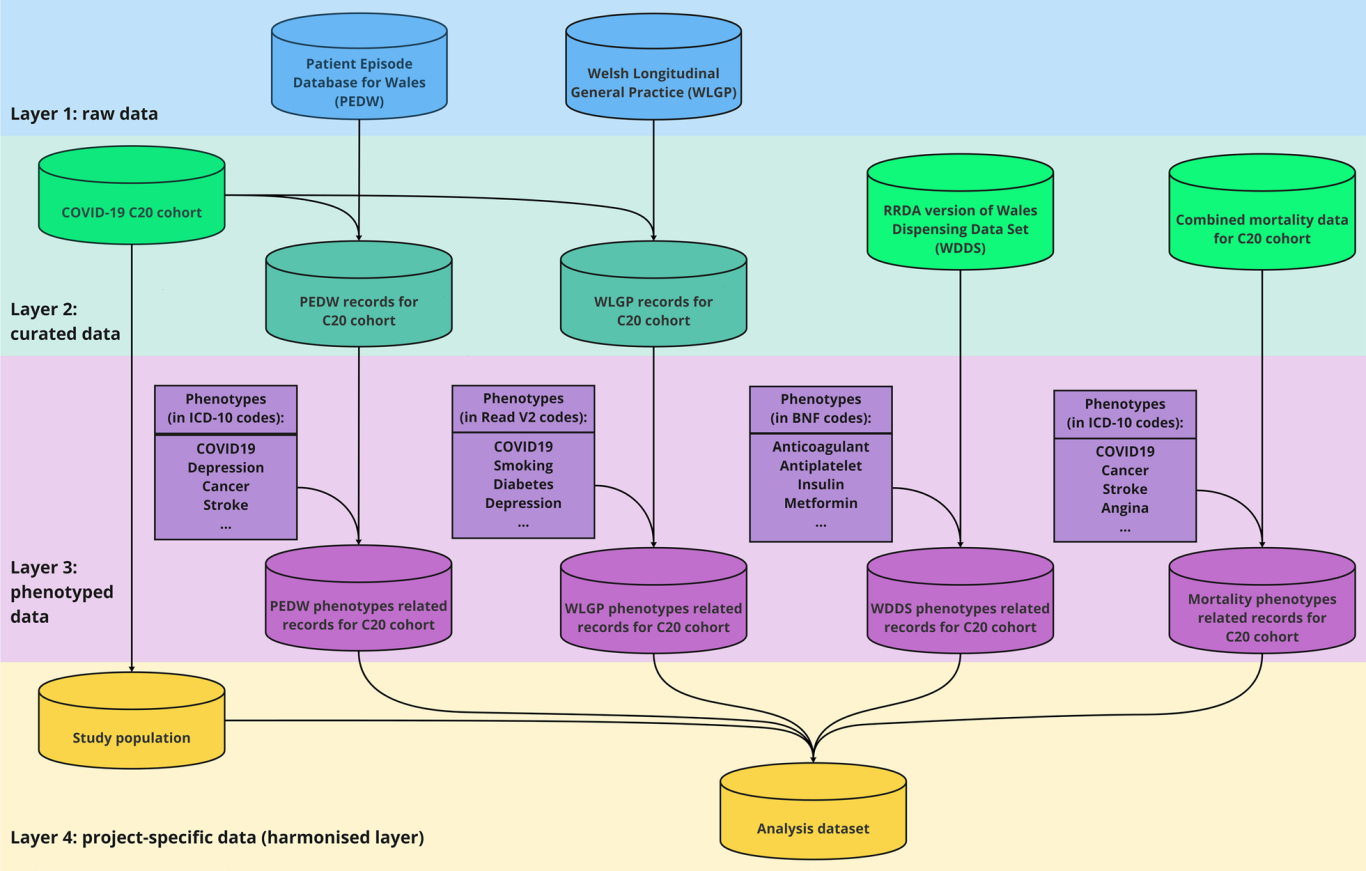
A simplified example of the four-layer data preparation process used to harmonise data within SAIL with data for England (within the NHS Digital TRE for England). Layer 1 consists of raw data sources in SAIL (e.g., primary care and secondary care data sources). Layer 2 includes Research Ready Data Assets (RRDAs) and generated curated version of raw data sources. Examples of RRDAs are the COVID-19 C20 cohort, combined mortality data for COVID-19 C20 cohort [47] and RRDA version of dispensing data [45]. In Layer 3, phenotypes related data are generated using Layer 2 data and phenotype code-lists. Many phenotype code-lists in the HDR UK Phenotype Library [12] have already been imported into SAIL (only a subset of phenotypes has been displayed for illustrative purposes). Finally, in Layer 4 fully harmonised project-specific data tables are derived from Layer 2 and 3 data

**Fig. 2:**
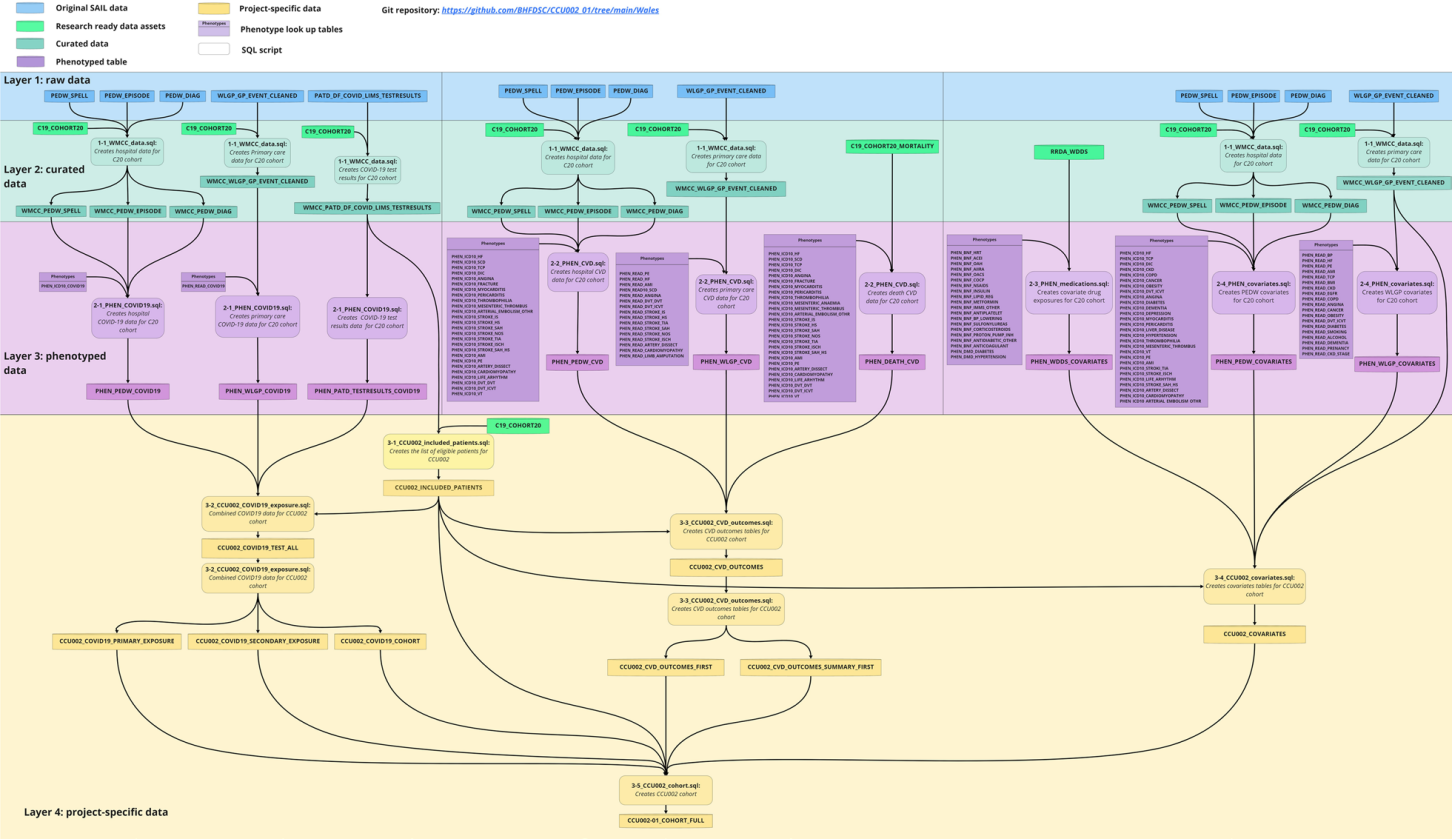
The four-layer data harmonisation process for Welsh data analysis used in [43]. See the GitHub repository [62] for the scripts

To address transparency and communication challenges, we have used best practices and rules established within the SAIL Databank for Wales and the NHS Digital TRE for England around naming conventions of files and folders and any database assets created and maintained. This ensures the effective organisation and understanding for users who may be actively working on a proposal or wish to learn or reuse existing components of the resources. Data visualisation has also been established to show the flow and layers of data preparation employed in delivering required data assets and research, which align with the underlying file names and locations. Figure 1 shows a simplified example of the four-layer process applied for some of these projects, and Fig. 2 illustrates the detailed version of the data harmonisation process used in [43].

### Layer 1 (raw data sources)

Layer 1 consists of raw data sources in SAIL which are available to all approved users conducting CVD-COVID-UK projects in a “read-only” database schema. Additional file 1 shows key details of all data sources currently available for CVD-COVID-UK projects within SAIL Databank and the NHS Digital TRE for England. All these raw data sources (apart from the data for ONS 2001 Census, and Congenital Anomalies Register and Information Services for Wales) are updated regularly within these TREs daily, weekly, fortnightly, or quarterly, depending on the data source. More information about these data sources and their meta-data can be found in the Health Data Research Innovation Gateway [44].

### Layer 2 (curated data)

There are two types of data tables in Layer 2 (derived from raw data sources in Layer 1). The first type are general purpose, pre-prepared, cleaned tables with derived columns, known as Research Ready Data Assets (RRDAs). These are generated from two or more raw data sources by applying quality checks, linkage, and pre-processing procedures [45]. The RRDAs are maintained by the Population Data Science group at Swansea University [46] and made available for several projects including CVD-COVID-UK.

An example of an RRDA is the COVID-19 “C20 electronic cohort” [47] which provides a population spine of 3.2 million Welsh residents alive and registered within the NHS in Wales from the 1st January 2020, including those who have moved into Wales or were born after 1st January 2020. Multiple demographic and healthcare data sources have been used to create the cohort (see [47] for more details), which is updated monthly. Columns cover information regarding demographics (e.g. age, sex, week of birth, date of death), residence (e.g. date moved in and out of Wales, residential anonymised linkage field, Lower-layer Super Output Area (LSOA, a geographic hierarchy in England and Wales used to estimate the characteristics of the people who live in a particular area) version 2011), and registration with primary care general practices. An equivalent entity in the NHS Digital TRE for England is the “key patient characteristics” table, which includes > 56 million individuals alive on 1st January 2020 and registered with an NHS general practice in England [32]. Primary care and hospital episode records (covering inpatient, outpatient, and emergency department episodes) have been combined prior to the index date of 1st January 2020 to define key characteristics, including sex and age. We have used these population denominators to derive harmonised variables for age, sex, date of death, and deprivation.

Another key RRDA in Layer 2 is the derived ethnicity data table in the SAIL Databank based on 26 data sources and harmonised into a national ethnicity spine for the population of Wales [48]. While ethnicity is usually considered a single variable in studies, each patient might have their ethnicity recorded once, many times, or never [9]. These codes might differ and even conflict for various reasons, including different categories being used across data sources and TREs [49]. Harmonised ethnic groups corresponding to the ONS categories (i.e. White, Mixed, Asian and Asian British, Black and Black British, and Other ethnic groups) are available in the ethnicity spine RRDA in SAIL and the key patient characteristics table in the NHS Digital TRE.

In addition to the RRDAs in Layer 2, we have generated a curated version of other raw data sources by applying initial data cleaning, which is common across projects. This cleaning process includes linking the data sources to two population spines for Wales, the C20 cohort and C16 cohort (a counterfactual and contextual comparative population spine consisting of the whole ~ 3 million population of Wales from the 1st January 2016 to 31st December 2019 [47], see Additional file 1 for more details), removing records whose unique anonymised identifier is missing, and applying some pre-processing procedures (e.g. removing records whose date is out of data coverage). Examples includes the curated version of the Welsh Longitudinal General Practice (WLGP), Patient Episode Dataset for Wales (PEDW), Outpatient Dataset for Wales (OPDW), and COVID-19 Test Results (PATD). All RRDAs and generated data tables in Layer 2 are updated monthly.

### Layer 3 (phenotyped data)

Layer 3 consists of phenotype-related data tables, called “PHEN DataSourceName PhenotypeName/Category”. These include all records related to a phenotype or group of phenotypes within a data table in Layer 2. Examples of Layer 3 data tables are “PHEN PEDW COVID19” (which includes all confirmed or suspected cases of COVID-19 in the curated version of PEDW data in Layer 2), and “PHEN PEDW CVD” (which contain all records related to cardiovascular diseases in the curated version of PEDW).

Data for secondary care systems such as hospital admissions, outpatient episodes, and mortality registers in Wales and England use the same clinical coding systems for diagnoses and causes of death, the International Classification of Diseases, 10th revision (ICD-10), and the Office of Population Censuses and Surveys codes version 4 (OPCS-4) for classification of hospital interventions and procedures clinical coding [44]. Therefore, phenotype code-lists developed using ICD-10 or OPCS-4 in either TRE could be used to generate the harmonised data tables related to these phenotypes.

Medication dispensed through community pharmacies are available in both SAIL Databank (for COVID-19 purposes) and the NHS Digital TRE for England. In Wales, dispensing data is available within the Welsh Dispensing DataSet (WDDS), which includes all NHS prescription items dispensed from all community pharmacies remunerated by NHS Wales, and is coded in the Dictionary of Medicines and Devices (DM+D). Work has been done in SAIL to also include British National Formulary (BNF) coding to this data through creating an RRDA version of WDDS [45]. This RRDA is linked to the C20 cohort and part of Layer 2 in our four-layer process. In England, the NHS Business Service Authority (NHSBSA) dispensing data includes prescriptions for all medicines dispensed in the community in England and is coded in BNF and DM+D. Therefore, any phenotype developed using DM+D or BNF in either TRE can be used.

However, this is not the case for other data sources, such as primary care general practice event data. In Wales, WLGP data is recorded in Read V2 codes, whilst in England, the General Practice Extraction Service (GPES) Data for Pandemic Planning and Research (GDPPR) is recorded in Systematized Nomenclature of Medicine Clinical Terms (SNOMED-CT) [44]. For primary care phenotypes, previously validated phenotypes in Read V2 code format from reputable sources such as CALIBER [37, 50] are used directly in SAIL, while conversion to Read V2 code format of phenotypes developed for use in the NHS Digital TRE for England in SNOMED-CT has been completed in collaboration with healthcare professionals and domain experts. Novel primary care phenotypes, such as COVID-19 diagnosis, have been developed in both SNOMED-CT and Read V2 in parallel (see Additional file 2). Furthermore, in the NHS Digital TRE for England, an assessment of comorbidity burden uses the number of disorders for each individual based on a SNOMED code-list obtained via an algorithm. The same approach could not be implemented with Read V2 codes (due to differences in the structure of this coding system compared with SNOMED-CT). Hence existing comorbidity indexes, such as the Charlson, Elixhauser and other available comorbidity indexes [51, 52], have been used to obtain this comorbidity burden variable for the Welsh population.

Emergency Department (ED) data, also known as Accident and Emergency (A&E) data, are available in Emergency Department Data Set (EDDS) within SAIL and in the Hospital Episode Statistics (HES) Accident and Emergency (HES-AE) data within the NHS Digital TRE for England. These data have their own coding system and variable format for diagnosis and treatment information. In addition, some Welsh hospitals use ICD-10 codes at a 3-character level in EDDS. So ED related phenotypes in these TREs have been harmonised following a detailed clinical review of mappings between these coding systems. For example, the diagnosis in HES-AE is a 6-character code consisting of diagnosis condition (n2), sub-analysis (n1), anatomical area (n2) and anatomical side (an1) [53]. While in EDDS, the diagnosis code has eight characters, consisting of diagnosis condition (an3), anatomical area (n3) and anatomical side (n2) [54]. So a phenotype such as lower limb fracture can be defined for each of these data sources using the related look up tables for diagnosis condition, sub-analysis (where applicable), and anatomical area and side.

Some methods developed based on specific data sources in one TRE might not apply to another TRE due to differences in the structure and fields contained within the corresponding data source(s) or the lack of similar data source between TREs. For example, the phenotypes defined for COVID-19 intensive care unit (ICU) admission, invasive and non-invasive ventilation for the NHS Digital TRE for England in [55] use code-lists in OPCS-4 for HES Admitted Patient Care (HES-APC) data source as well as specific fields in the following data sources (and not clinical coding): HES for Adult Critical Care (HES-CC) and COVID-19 hospitalisation information from COVID-19 Hospitalisations in England Surveillance System (CHESS). In SAIL, hospital interventions and procedures are recorded in PEDW in OPCS-4, and so phenotypes coded in this coding system can be used in SAIL. However, the intensive care and critical care data in SAIL (available in Critical Care Data Set (CCDS), and ICNARC—Intensive Care National Audit and Research Centre data (ICNC)) are different, and independent approaches [56] have been developed with a similar goal to identify and derive the outcomes needed.

Unified phenotypes related to COVID-19 polymerase chain reaction (PCR) tests, lateral flow tests, and vaccination have been defined using similar data sources in these TREs. In addition, based on the project’s need, phenotypes specific to Wales Results Reporting Service (WRRS, which contains all pathology laboratory results in Wales) have also been developed [39]. Examples are phenotypes (including test codes, their description, unit, and reference ranges) for influenza, pneumonia and other respiratory tract infections.

All phenotypes are documented and uploaded to the Health Data Research UK Phenotype Library [12], and BHF DSC GitHub repository [57] upon completion, signoff, and implementation as part of submitted published work. All generated data tables in Layer 3 are updated following the monthly update of Layer 2 data tables.

### Layer 4 (project-specific data tables)

Finally in Layer 4, project-specific data tables are created containing fully harmonised data tables as structured and formatted in both TREs. That is, all data table names, column names, and applicable values and ranges are the same between TREs. For example, demographic categories and outcomes of interest such as sex, age, ethnic groups, smoking status, or cardiovascular-related outcomes are the same for use in research analyses. Also due to the scale of geography and population size of Wales and England, Wales has been considered one region when combining results with England, which has nine defined regions (North West, North East, East of England, London, East Midlands, West Midlands, Yorkshire and the Humber, South East, South West). When evaluating the impact of socioeconomic factors, the Welsh Index of Multiple Deprivation [58] and the English Index of Multiple Deprivation [59] have been used with consideration of the differences between the respective indexes, as the quintiles are not directly comparable between them. Therefore, any analytical pipeline developed in one TRE can be applied to the other with minimal/no change, and then results from these TREs can be combined across nations using appropriate meta-analysis methods.

Initial quality checks and descriptive statistics (e.g., frequencies, median, mean, standard deviation, and ranges) were used to assess the quality of the process and project-specific variables and to compare the consistency (distribution and missing values) of the harmonised data with corresponding data for England. Where required, researchers from both TREs engaged in discussions to understand any potential causes of inconsistencies and to clarify potential solutions.

**Fig. 3:**
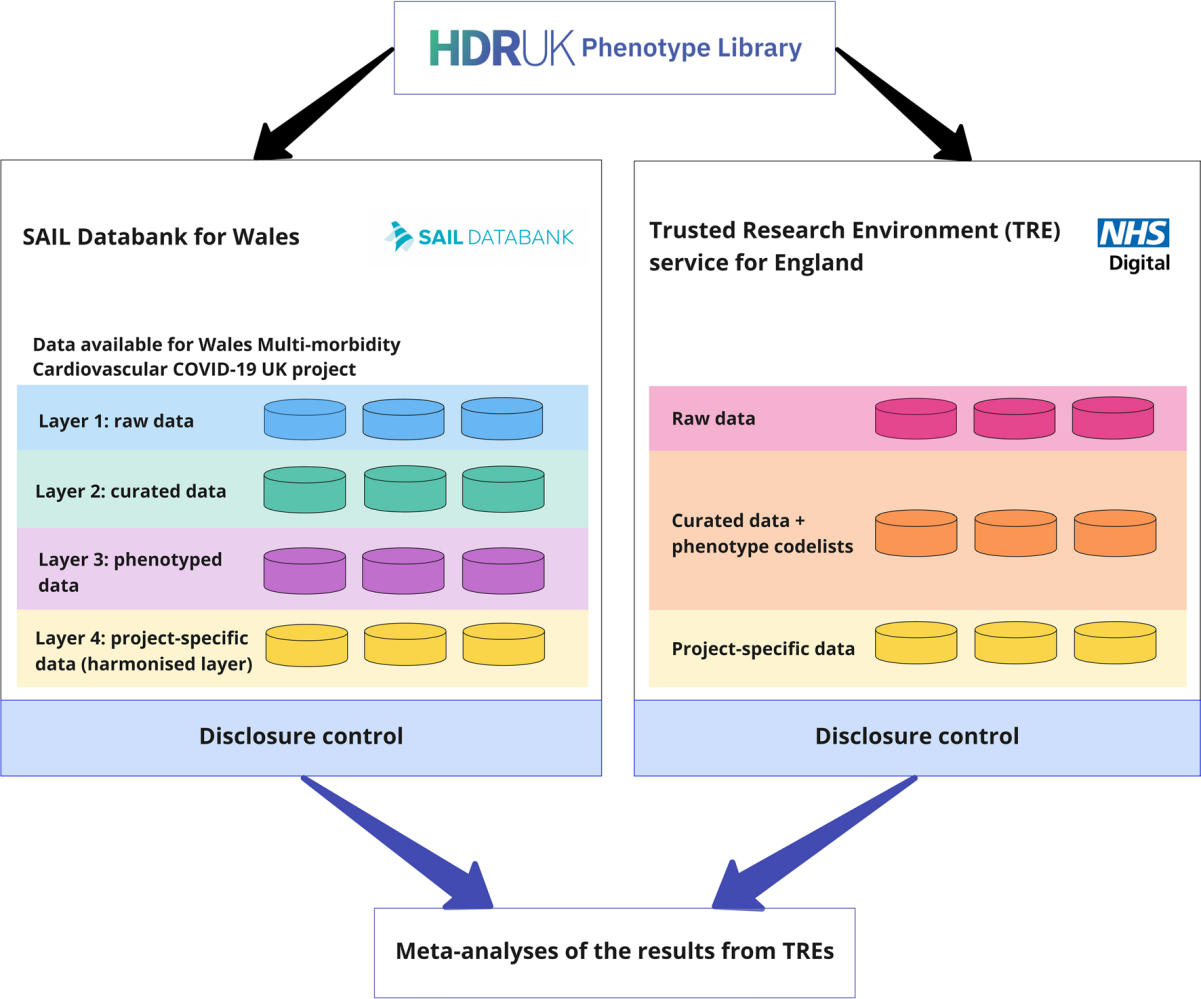
The process for combining analyses results from SAIL (Wales) and NHS Digital TRE for England. The SAIL Databank for Wales and the NHS Digital TRE for England provide a secure remote data access system and analysis environment. Many phenotype code-lists in the HDR UK Phenotype Library [12] have already been uploaded/imported in these TREs. Approved researchers within each TRE can access data and phenotype code-lists, and perform analyses in the TRE. Then the results of analyses from these TREs can be combined (using meta-analysis) outside of the TREs once approved through the TREs disclosure control process, with phenotype code-lists and code accessible outside the TREs, and a copy of what is needed imported/exported from the TREs as required through standard disclosure control processes

Figure 3 shows the process for combining results of analyses from SAIL and NHS Digital TRE for England for CVD-COVID-UK projects. In SAIL, disclosure control through file out requests do not permit outputs that would intentionally or unintentionally break the privacy-protection of the anonymised data, primarily handled through a small number policy (< 5 as standard, and < 10 when using any data obtained through the DEA including the ONS 2011 Census data), which entails that the results are considered disclosive, and therefore should be suppressed. Very similar processes are used for disclosure control in the NHS Digital TRE. So, if any results requested out of each TRE (which are required for meta-analysis and/or to be included in the final output(s)) fall below these thresholds, then there will be an issue as unadjusted analysis should be excluded, and counts < 5 for adjusted analysis should be masked. A solution for this issue could be composite outcomes at a different or higher level of aggregation.

We note that the software used for data preparation, generating analytical outputs, visualisations, and results have been different in these TREs due to the availability of different software tools in the TREs. For example, for population-wide analyses, the size of the data in the NHS Digital TRE for England requires distributed computing. So Apache Spark (a data processing engine for distributed computing which sits between the data source and the analysis tool) is provided in this TRE to run SQL queries, and can be utilised using Spark SQL or Python query tools such as PySpark [60]. In SAIL, similar tools (such as Eclipse and Jupyter notebooks) can be used to run SQL queries. For more details about available analytical and version control tools in the SAIL and the NHS Digital TRE for England see [7, 61].

All data tables generated as part of the harmonisation process include individual-level details. Hence, these tables are only accessible within the SAIL Databank TRE. In order to access these resources, researchers working on the CVD-COVID-UK program will require to submit their proposals to the SAIL via (https://www.saildatabank.com/application-process), and all applications are reviewed by an independent Information Governance Review Panel (IGRP). The IGRP considers each project to ensure proper and appropriate use of SAIL data. When access has been granted, it is gained through a privacy protecting safe haven and remote access system, referred to as the SAIL Gateway. Further details of this process can be found on the SAIL Databank website (https://saildatabank.com/).

All SQL and R scripts used to generate data tables in Layers 2,3 and their associated documentation, as well as all scripts used to derive project-specific data tables (in Layer 4) and related meta-data are available in GitLab within the SAIL Gateway, and made publicly available via the BHF DSC GitHub repository [57] following completion of the project.

Finally, although this harmonisation process has been implemented as part of the CVD-COVID-UK programme to enable cross-nation COVID-19 related analysis in England and Wales, the data harmonisation methodology, data curation and linkage techniques, phenotypes definition, and derivation of analysis variables can be generalised and used by other projects using the SAIL Databank and replicated across other TREs across the UK with similar data sources.

## Results

**Fig. 4:**
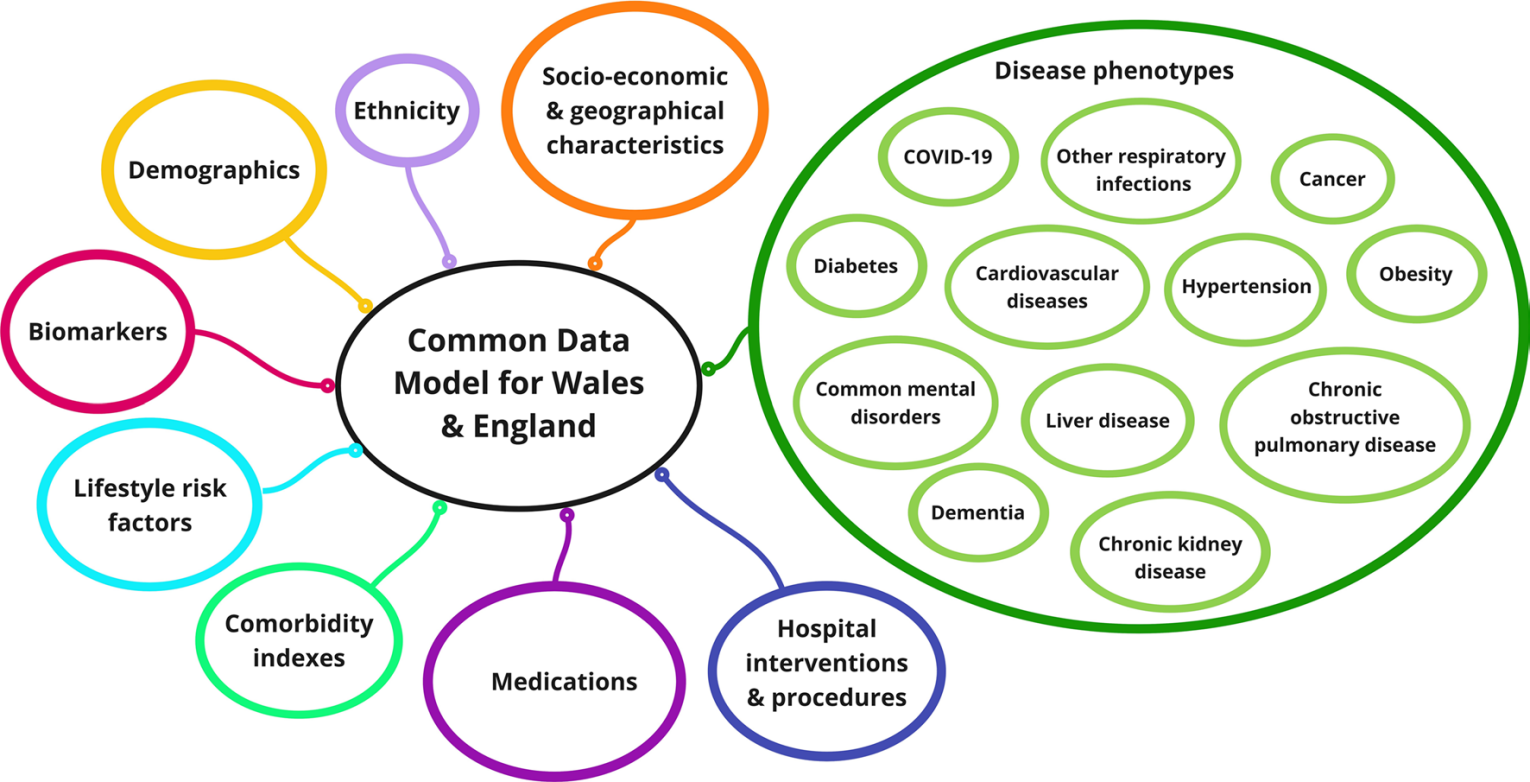
Categories of harmonised variables for population of Wales and England

Using linked individual-level EHR, demographic and administrative data, we harmonised approximately 100 analysis variables for the population of Wales with corresponding data for England, as part of the CVD-COVID-UK programme. Harmonised variables were grouped into the following categories: demographic variables (e.g., age, sex, date of death, and size and the average age of general practices on 1st January 2020), ethnic group, socio-economic and geographical characteristics (deprivation, LSOA 2011, and region), disease phenotypes including COVID-19 related and CVD related phenotypes, biomarkers (body mass index, and blood pressure), lifestyle risk factors (smoking status, and alcohol consumption), comorbidity indexes (e.g., Charlson and Elixhauser comorbidity indexes), hospital interventions and procedures (e.g., ICU admission, invasive and non-invasive ventilation), medications (e.g., angiotensin-converting enzyme inhibitors, antiplatelet drugs, lipid regulating drugs, and anticoagulants), and other variables (e.g., number of unique dispensed medications, and primary care consultation rate). Variables categorised as disease phenotypes or comorbidity indexes were extracted from primary care and secondary care data, mortality data, and laboratory results, using phenotypes coded in ICD-10 or Read V2, or phenotypes specific to a data source in SAIL (e.g., phenotypes for ED data or critical care data, see Additional file 1). Biomarkers and lifestyle risk factors were derived from primary care data using code-lists in Read V2 terms. Variables in the medication category were created using dispensing data and phenotypes coded in BNF. Hospital interventions and procedures were identified using appropriate fields from secondary care data in addition to OPCS-4 procedure codes. Figure 4 shows categories of harmonised variables, and Additional file 1 provides an overview of data sources and their coding system.

The reproducible approach for data harmonisation described is being used in CVD-COVID-UK projects, leading to peer-review publications. In these projects, the same analytical pipeline for each project was applied within each TRE, and results were combined via meta-analysis across nations. For each project, the protocol, code-lists used for phenotypes, and SQL and R codes are available on GitHub together with any published output. Some examples include:

- SARS-CoV-2 infection and risk of venous thromboembolism and arterial thrombotic events in England and Wales (project reference “CCU002_01” in [30]): (published paper [43], all code and phenotypes used to produce this paper are available in [62]). Figure 2 shows the data harmonisation process used in [43].
- COVID-19 vaccination and disease and the risks of myocarditis and pericarditis (project reference “CCU002_03” in [30]): Code and phenotypes used in this study are available in [63].
- Assessing cardiovascular disease impact through medicines (project reference “CCU014_01” in [30]): The WDDS RRDA was used in [64] to harmonise Wales dispensing data in SAIL with corresponding data in the NHS Digital TRE for England. Code and phenotypes used in this study are available in [65].

## Discussion

The COVID-19 pandemic has highlighted the need to implement efficient approaches that enable multi-nation analytics across different TREs. We have addressed this challenge by creating a national harmonisation method which to date has served six projects across England and Wales and can be scaled up and expanded to many more in the future.

The harmonisation method has been implemented as a four-layer process to achieve reproducibility and scalability, starting from raw data in the first layer, followed by curated data in the second layer, phenotyped data in the third layer, and finally project-specific data in the fourth layer. The key benefits of data harmonisation using such a reproducible approach are as follows. Firstly, it makes replicating the code much easier, whether revisiting an old project, making revisions following peer review, or extending the research. For example, when changing or extending the study period or inclusion/exclusion criteria, only certain tables in specific layers need to be modified and updated, while others remain unchanged. Secondly, transparency can be easily reached by such a reproducible approach. This reduces risk of errors during study development through allowing cross-checking of results between TREs as well as aiding external validation of results. Thirdly, initial data cleaning processes performed for SAIL data sources in Layer 2 are similar across projects, increasing the transferability of learning and enabling new studies to start more quickly. Phenotypes added to data tables in Layer 3 are also available for all new studies to use. Therefore, data tables generated in Layers 2 and 3 allow researchers to start from these layers and derive project-specific data tables for their project. This removes the need for initial data cleaning, accelerates the data preparation process, and increases efficiency. Fourthly, methods used for generating harmonised project-specific data items in Layer 4 and methods used for combining Welsh and English results are useful and reusable for future projects studying the population of both nations. Lastly, new phenotyped data tables can be easily added to Layer 3, or existing phenotyped data can be updated or expanded upon revision or addition of a phenotype (these tables are created in a wide format to allow the addition of new fields). All the points above illustrate that this methodology and the respective layers can be iteratively updated and are scalable.

Data harmonisation across TREs has limitations. Data used to derive harmonised variables are limited to what is available within the respective TREs. For example, some data sources available in one TRE might not be available in others (see Additional file 1 for a comparison of available data sources currently in the SAIL Databank and the NHS Digital TRE for England). Other limitations are associated with the general limitations of routine health data. Healthcare systems generate large amounts of routine data for clinical and administrative purposes in settings such as hospitals, laboratories, general practices and pharmacies. However, as routine health data are not primarily collected for research, the usability of these data presents several limitations. These limitations include potential incompleteness, inconsistency over time and between systems with differing coding systems, varying rates of data accuracy over time and between systems, and duplicate records.

### Lessons learnt and future opportunities

Data harmonisation is a time-consuming process and requires technical and scientific investments. Here we outline lessons learnt and best practices based on our experience to facilitate this process:

1. Where a protocol is developed based on available data sources in one TRE and then extended to other TREs, it may not reflect the potential or limitations of the data sources available across all of the TREs. Therefore, as suggested in the Maelstrom guidelines [16], before the harmonisation process begins, it is necessary to develop a project-specific protocol reflecting the strengths and limitations of data harmonisation and combining results from these TREs.
2. The variables for harmonisation should be clearly defined, including their specific nature, format and, where necessary, their acceptable level of heterogeneity. Furthermore, creating early summary statistics on the cohort generated in each TRE to compare numbers between the two/multiple populations is useful to see if demographic and disease counts are similar or demonstrate expected differences. This provides confidence in the harmonisation of data items.
3. It is important to be consistent with naming data items, data tables, files, folders and even objects in the analyses across TREs. Consistent naming conventions helps to order files easily and makes the contents and relationships among data items, tables, and elements of the analyses understandable and searchable.
4. Data cleaning, merging, and transforming rules should be done via scripts, not manually. This is particularly important when multiple research team members have access to the data and make modifications. Coding all the relevant rules can be challenging but saves time in the long term.
5. Closely documenting the processes used for data harmonisation is necessary for transparency, reproducibility and sustainability. All derivations from the data sources should be documented with a clear description of all the data cleaning rules and the rationale for deriving new variables.
6. Tools used for documentation and visualisation of the data preparation process (such as Miro, and R Markdown) facilitate communication by visualising and explaining complex relationships, dependencies and levels of preparation in the data and analytical pipeline so that both the team completing the steps and the users that utilise the output from the pipeline have a transparent and clear understanding of the end-to-end process, and where to access various code and files at the various stages and layers. This includes version control of all statistical analyses and data management code, documentation, and other files and generated data and outputs.

Additional opportunities exist to refine further and develop the pipelines, methods, and approaches within the CVD-COVID-UK consortium. These include but are not limited to: the development of new RRDA’s, which encapsulate specific data sources or combinations of data sources around specific themes or requirements that other users and future research studies would benefit from accessing as a ready-to-use data table rather than just the code or components of the code; establishing further phenotypes and harmonisation strategies, including code and notebook templates for analytical and statistical requirements; expanding the harmonisation to additional data sources within the existing TREs and accessing and deploying the methodologies into new TREs around the UK and worldwide.

## Conclusion

We implemented a collaborative, transparent, and reproducible process to generate valuable harmonised EHRs for Wales to be used (within the CVD-COVID-UK programme) for research on COVID-19 and its relationship with CVD for the population of Wales and England. This paper describes challenges for harmonising EHRs between TREs and the harmonisation process used to address these challenges for SAIL Databank and the NHS Digital TRE for England, as well as best practices and recommendations for retrospective data harmonisation across these TREs. More broadly, it provides an example of how large-scale multi-national collaborations can successfully implement and document retrospective harmonisation to generate comparable demographic and health indicators.

## Supplementary Information

**Additional file 1.** Microsoft Word (.doc)—Summary of all data sources available for CVD-COVID-UK projects in the SAIL Databank and NHS Digital TRE for England.

**Additional file 2.** Microsoft Word (.doc)—Code-lists in SNOMED-CT and Read V2 for COVID-19 diagnosis in primary care data.

## Data Availability

The data used in this study are available in the SAIL Databank at Swansea University, Swansea, UK, but as restrictions apply they are not publicly available. All proposals to use SAIL data are subject to review by an independent Information Governance Review Panel (IGRP). Before any data can be accessed, approval must be given by the IGRP. The IGRP gives careful consideration to each project to ensure proper and appropriate use of SAIL data. When access has been granted, it is gained through a privacy protecting safe haven and remote access system referred to as the SAIL Gateway. SAIL has established an application process to be followed by anyone who would like to access data via SAIL at https://www.saildatabank.com/application-process. The data used in this study are available in NHS Digital’s TRE for England, but as restrictions apply they are not publicly available (https://digital.nhs.uk/coronavirus/coronavirus-data-services-updates/trusted-research-environment-service-for-england). The CVD-COVID-UK/COVID-IMPACT programme led by the BHF Data Science Centre (https://www.hdruk.ac.uk/helping-with-health-data/bhf-data-science-centre/) received approval to access data in NHS Digital’s TRE for England from the Independent Group Advising on the Release of Data (IGARD) (https://digital.nhs.uk/about-nhs-digital/corporate-information-and-documents/independent-group-advising-on-the-release-of-data) via an application made in the Data Access Request Service (DARS) Online system (ref. DARS-NIC-381078-Y9C5K) (https://digital.nhs.uk/services/data-access-request-service-dars/dars-products-and-services). The CVD-COVID-UK/COVID-IMPACT Approvals & Oversight Board (https://www.hdruk.ac.uk/projects/cvd-covid-uk-project/) subsequently granted approval to this project to access the data within NHS Digital’s TRE for England and the Secure Anonymised Information Linkage (SAIL) Databank. The de-identified data used in this study were made available to accredited researchers only. Those wishing to gain access to the data should contact bhfdsc@hdruk.ac.uk in the first instance.

## Abbreviations

BHF: British Heart Foundation
BNF: British National Formulary
CCDS: Critical care data set
CDM: Common data models
CHESS: COVID-19 Hospitalisations in England Surveillance System
CVD: Cardiovascular disease
DEA: Digital Economy Act
DM+D: Dictionary of medicines and devices
DSC: Data Science Centre
EHR: Electronic health record
ED: Emergency department
EDDS: Emergency department data set
GPES: General Practice Extraction Service data
GDPPR: GPES Data for Pandemic Planning and Research
HDR UK: Health Data Research UK
HES: Hospital episode statistics data
HES-AE: HES accident and emergency data
HES-APC: HES admitted patient care
HES-CC: HES adult critical care data
ICD-10: International Classification of Diseases, 10th revision
ICNARC: Intensive Care National Audit and Research Centre
ICNC: ICNARC dataset
ICU: Intensive care unit
LSOA: Lower-layer Super Output Area
NHS: National Health Services
NHSBSA: NHS Business Service Authority
OMOP: Observational Medical Outcomes Partnership
ONS: Office for National Statistics
OPCS-4: Office of Population Censuses and Surveys codes, version 4
OPDW: Outpatient dataset for wales
PATD: COVID-19 test results
PCR: Polymerase chain reaction
PEDW: Patient Episode Database for Wales
RRDA: Research ready data asset
SAIL: Secure Anonymised Information Linkage (Databank)
SNOMED-CT: Systematized Nomenclature of Medicine Clinical Terms
SQL: Structured Query Language
TRE: Trusted Research Environment
WDDS: Welsh dispensing dataset
WLGP: Welsh Longitudinal General Practice data
WRRS: Wales Results Reporting Service
