# Supplementary material for: Harmonising electronic health records for reproducible research: challenges, solutions and recommendations from a UK-wide COVID-19 research collaboration
Source: BMC Med Inform Decis Mak. 2023 Jan 16;23:8. doi: 10.1186/s12911-022-02093-0 (PMC9842203; doi:10.1186/s12911-022-02093-0)
Supplement: Supplementary file 1 — Additional file 1. Microsoft Word (.doc)—Summary of all data sources available for CVD-COVID-UK projects in the SAIL Databank and NHS Digital TRE for England. [file 12911_2022_2093_MOESM1_ESM.docx]

**Additional file 1: Summary of all data sources available for CVD-COVID-UK projects in the SAIL Databank and NHS Digital TRE for England.**

|  | **SAIL Databank for Wales** | | | | | **NHS Digital TRE for England** | | | | |
| --- | --- | --- | --- | --- | --- | --- | --- | --- | --- | --- |
|  | **Resource name** | **Contents** | **Coverage** | **Coding** | **Refreshed** | **Resource name** | **Contents** | **Coverage** | **Coding** | **Refreshed** |
| **Primary care** | Welsh Longitudinal General Practice (WLGP), | Personal characteristics and all interactions with primary care services including symptoms, diagnoses, investigations, prescriptions and referrals to tertiary and secondary care | 1990-01-01 to 2022-03-25 | READ V2 | Monthly | General Practice Extraction Service (GPES) Data for Pandemic Planning and Research (GDPPR) | Personal characteristics, COVID-19 activity, contraindications, diagnoses, prescribed medications, management and treatment, screening, vaccination and immunisations, review and monitoring, and tests. Of over 900,000 SNOMED codes in the UK, only around 36,000 codes are used within the GDPPR. The data contain records of active and current GP registrations with any code relevant to pandemic planning and research, as well as deceased patients with a date of death on or after 1^st^ November 2019 | 1950-01-01 to  2022-06-29^1^ | SNOMED-CT | Update from source: Fortnightly (approximately one week lag)    TRE update: Monthly |
|  | General Practice COVID Daily (GPCD) | Same as WLGP | 2000-01-01 to 2022-04-01 | READ V2 | Daily |  |  |  |  |  |
| **Secondary care (hospital admissions, emergency department, outpatients, critical care)** | Patient Episode Database for Wales (PEDW) | All inpatient and day-case activity undertaken in NHS Wales plus data | 1995-04-01  to 2022-08-31 | ICD-10  OPCS-4 | Weekly | Hospital Episode Statistics Admitted Patient Care  (HES-APC) | Details of episodes of care during an inpatient hospital admission | 1997-01-01 to  2022-03-31^1^ | ICD-10  ICD-9  OPCS-4 | TRE update: Monthly (approximately 6 weeks lag) |
|  | Emergency Department Data Set (EDDS) | Attendance and clinical information for all Accident and Emergency attendances | 2008-04-05 to 2022-09-01 | ED specific coding | Monthly | Hospital Episode Statistics Accident & Emergency (HES-AE) | Details of individual Accident and Emergency (A&E) attendances and major incidents at all A&E departments, walk-in centres, and minor injury units in England | 2007-01-01 to  2022-03-31^1^ | A&E diagnosis classification code    ICD-10  Read V2 | TRE update: Monthly (approximately 6 weeks lag) |
|  | Emergency Department Dataset Daily (EDDD) | Same as EDDS | 2010-07-22 to 2022-09-02 | ED specific coding | Daily |  |  |  |  |  |
|  | Outpatient Dataset for Wales (OPDW) | Attendance information for all hospital outpatient appointments | 1991-01-01 to 2022-09-01 | ICD-10 | Monthly | Hospital Episode Statistics Outpatient (HES-OP) | Details of individual records of outpatient appointments, including the type of outpatient consultation, appointment dates, details of the speciality under which the patient was treated, waiting times, and referrals | 2003-01-01 to  2022-03-31^1^ | ICD-10 | TRE update: Monthly (approximately 6 weeks lag) |
|  | Outpatient Referral Dataset (OPRD) | Data on Outpatient referrals from primary care | 2009-04-01 to 2022-09-01 | - | Monthly | - | - | - | - | - |
|  | ICNARC – Intensive Care National Audit & Research Centre (ICCD) | All ICU admissions | 2020-03-12 to 2022-08-03 | Text description | Quarterly | Intensive Care National Audit & Research Centre (ICNARC) | Intensive care admissions for critically ill patients with confirmed COVID-19 | 2020-02-05 to  2021-12-20 | ICNARC Coding Method (ICM) | Update from ICNARC: Weekly    TRE update:  Once so far in Feb 2022 |
|  | ICNARC COVID only admissions (ICNC) | All ICU COVID-19 admissions | 2021-01-19 to 2022-08-03 | Text description | Monthly |  |  |  |  |  |
|  | Critical Care Data Set (CCDS) | All critical care admissions | 2007-01-01 to 2022-09-01 | - | Quarterly | Hospital Episode Statistics Critical Care  (HES-CC) | Details of patients and their episodes for treatment and critical care (intensive care or high dependency care) at NHS hospitals in England | 2008-01-01 to  2022-03-31^1^ | Critical Care Activity Code    OPCS-4 | TRE update: Monthly (approximately 6 weeks lag) |
|  |  |  |  |  |  | Critical Care Augmented Care Period (ACP) | A retired dataset of HES critical care activity introduced in 1996, containing data of intensive care or high dependency units (ICU/HDU) episodes. It was mandatory for all acute Trusts in England to collect this dataset from 1^st^ October 1997 to 1^st^ April 2006 when it was replaced by the Critical Care Minimum Dataset (CCMDS). CCMDS is a part of the ICNARC dataset. | 1997-05-01 to 2007-10-01 | Refer to HES-APC and HES-CC | One-off |
|  | - | - | - | - | - | Uncurated Low Latency Hospital Data (HES-APC, HES-OP, HES-CC) | Uncurated, unadjusted, less assured derived, and non-derived pseudonymised SUS data related to HES-APC, HES-OP, and HES-CC provisioned for secondary purposes | HES-APC: 2019-01-01 to 2022-05-30^1^    HES-OP: 2019-01-01 to 2022-04-25^1^ | Same as HES-APC, HES-OP, and HES-CC | Update from source: Up to one month    TRE update: Monthly |
|  | - | - | - | - | - | COVID-19 SARI-Watch (formerly CHESS) | Demographic, risk factor, treatment, & outcome information for patients admitted to the hospital with a confirmed COVID-19 diagnosis | 2019-12-01 to  2022-06-29 | - | Update from source: Daily    TRE update: One to two months |
|  | - | - | - | - | - | Secondary Uses Services (SUS) | Collection of curated data required for purposes other than direct clinical care such as planning health care, supporting payment, commissioning, public health, clinical audit and governance, performance improvement, medical research, and policy development. The SUS Data in TRE is related to HES-APC hospital spells only. | 2019-06-01 to  2021-12-22^1^ | ICD-10  OPCS-4 | Update from source: Monthly    TRE update: One to two months |
| **Population denominators** | RRDAs C16 and C20 cohorts (C16 cohort includes all individuals living in Wales and known to the NHS on the 1^st^ January 2016 with follow-up to 31^st^ December 2019, and C20 cohort consists of all Welsh residents alive and living in Wales on the 1^st^ January 2020) | Demographic data such as age, sex, date of death, date of movement out of Wales, residential anonymised linkage field at cohort inception, Lower layer Super Output Area (LSOA) 2011 boundaries (small statistical areas containing around 1500 people) and their mapped Welsh Index of Multiple Deprivation (version 2019) quintile s, Welsh health board of residence, and urban/rurality categories | C16: 2016-01-01 to 2019-12-31    C20: 2020-01-01 to 2022-05-31 | - | Monthly | Key patient characteristics table | A table with pseudonymised record identifiers as the index of rows containing key characteristics per patient, including birth date, death date, sex, age, and ethnicity. It should be noted that IDs in this table include duplicated and inactive unregistered patients; hence, the dataset's size does not represent the population denominator. | Depending on the reference dataset. For example, if only the pseudonymised IDs in GDPPR are considered, then the coverage would be the same as the GDPPR. | - | TRE update: Monthly |
| **Mortality** | RRDA mortality for C16 and C20 cohorts, derived from Annual District Death Extract (ADDE), Annual District Death Daily (ADDD), Welsh Demographic Service Dataset (WDSD), Consolidated Death Data Source (CDDS) | Combined mortality data (including date of death, date of birth, sex, all causes of death) | For C16 cohort: 2016-01-01 to 2019-12-31    For C20 cohort: 2020-01-01 to 2022-09-01 | ICD-10 | Monthly | Civil Registration-Deaths (Office for National Statistics) | Date of death, the underlying cause of death, other diagnoses, sec, district, subdistrict, and place of death (code, establishment, and type) | 1993-01-02 to  2022-06-18 | ICD-10 | TRE update: Monthly |
| **Laboratory tests**  **for COVID-19** | Pathology data COVID-19 Daily (PATD ) - Pillar 1, 2^2^ | Test results from Laboratory Information Management System for Pillar 1 and 2 COVID19 tests (coronavirus SARS CoV2 PCR and coronavirus PCR tests) | 2020-02-03 to 2022-09-02 | - | Daily | COVID-19 Second Generation Surveillance System (SGSS) (Pillar 1, 2 first positive results only) | Demographic and diagnostic information from laboratory test reports for the first positive Pillar 1 swab testing in Public Health England (PHE) labs and NHS hospitals and Pillar 2 Swab testing in the community | 2020-01-01 to  2022-06-29 | - | Update from source: Daily    TRE update: Monthly |
|  |  |  |  |  |  | COVID-19 UK Non-hospital Antigen Swab Testing Results - Pillar 2 | The results of non-hospital antigen testing, details of the testing centre and laboratory, test kit type, and patient’s general information, including occupation and working/studying status | 2020-01-01  2022-06-29 | - | TRE update: Monthly |
|  | PATD Pathology data COVID-19 Daily (PATD) - Pillar 3^2^ | Test results from Laboratory Information Management System for Pillar 3 COVID19 tests (coronavirus SARS CoV2 PCR and coronavirus PCR tests). | 2020-02-03 to 2022-09-02 | - | Daily | COVID-19 UK Non-hospital Antibody Testing Results - Pillar 3 | Positive, negative, and void results of the non-hospital antibody testing results, testing centre and laboratory details, and patient’s general information, including occupation and working/studying status | 2020-08-26 to  2022-06-25 | - | TRE update: Monthly |
|  | COVID-19 Test, Trace and Protect (CTTP) | Information recorded via the COVID-19 Test Trace and Protect programme | 2021-06-01 to 2022-09-02 | - | Daily | - | - | - | - | - |
|  | COVID-19 Shielded People list (CVSP) | List of high-risk people advised to self-isolate during COVID-19 pandemic | 2020-05-12 to 2022-08-02 | - | Daily | - | - | - | - | - |
|  | Variant strain data (CVSD) | Information about SARS-CoV-2 variants of concern and variants under investigation in Wales (including phylogenetic and mutational information about samples) | 2021-05-07 to 2021-11-18 | - | Weekly | - | - | - | - | - |
| **COVID-19 vaccination** | RRDA^3^ version (de-duplicated, cleaned version) of COVID Vaccine Data (CVVD) | All patients and vaccinations administered or planned for COVID-19, in or funded by the NHS | 2020-12-08 to 2022-09-02 | - | Weekly | COVID-19 vaccination status | Data related to COVID-19 vaccination status, including patient demographics, source organisation, vaccination details, and vaccine batch | 2020-12-08 to  2022-06-26 | - | TRE update: Monthly |
|  | - | - | - | - | - | COVID-19 vaccination adverse reactions | Patient demographics, source organisation, and details of any adverse reactions which occur within the first fifteen minutes after administration of the coronavirus (COVID-19) vaccination | 2020-12-08 to  2022-06-25 | SNOMED-CT | TRE update: Monthly |
| **Prescribing/ Dispensing** | RRDA^3^ version (de-duplicated, cleaned, linked and BNF-mapped version ((38))) of Wales Dispensing DataSet (WDDS) | All NHS prescription items dispensed from all community pharmacies | 2016-01-01 to 2022-07-28 | DM+D  BNF | Monthly | NHS Business Service Authority (NHSBSA) dispensed medicines in primary care data | Patient-level data and information of drugs (name, strength, substance, quantity) related to medicines dispensed and claimed after prescription in primary care, including general practice, community clinics, hospital clinics, dentists, and community nursing services | 2018-04-01 to  2022-02-01 | DM+D  BNF | Update from source: Monthly    TRE update: Monthly |
|  | - | - | - | - | - | Prescribed medication: Refer to GDPPR | Prescribed medication: Refer to GDPPR | Prescribed medication: Refer to GDPPR | Prescribed medication: Refer to GDPPR | Prescribed medication: Refer to GDPPR |
|  |  |  |  |  |  | Electronic Prescribing and Medicines Administration- EPMA | The details of medicines prescribed and administered to patients as extracted from secondary care NHS trusts in England using electronic Prescribing and Administration system (ePMA) supplied by CareFlow Medicines Management (CMM).  The sub-categories of the dataset are data for a) prescription and b) administration, each containing a unique hash index per record. The prescription sub-category is linked with a1) prescription dataset coded in DM+D, a2) prescription dosage, and a3) active ingredient of the medicine. The administration sub-category is further linked with b1) administration data coded in DM+D and b2) the dosage of the administered medication. | 2020-30-11 to  2022-06-26 | DM+D | Update from source: Daily    TRE update: Monthly |
| **Laboratory results for pathology** | Wales Results Reporting Service (WRRS) | Pathology laboratory results (Haematology, Biochemistry, immunology, Cervical Cytology, Infection Control Services) | 1992-06-01 to 2022-08-22 | WRRS specific coding | Fortnightly | - | - | - | - | - |
| **Birth and maternity** | Annual District Birth Extract (ADBE) | Office for National Statistics (ONS) register of all births in Wales | 1996-01-01 to 2022-07-01 | ICD-10 | Quarterly | - | - | - | - | - |
|  | National Community Child Health database (NCCH) | The Child Health System in Wales; includes birth registration and monitoring of child health examinations and immunisations | 1989-01-01 to 2022-08-02 | ICD-10 | Quarterly | - | - | - | - | - |
|  | Maternity Indicators Dataset (MIDS) | All data relating to the woman at initial assessment and to mother and baby (or babies) for all births | 2014-04-01 to 2022-09-01 | - | Monthly | - | - | - | - | - |
|  | Congenital Anomaly Register and Information Services for Wales (CARS) | Information about any foetus or baby who has or is suspected of having a congenital anomaly and whose mother is normally resident in Wales at time of birth | 1998-01-01 to 2019-12-16 | ICD-10 | Yearly | - | - | - | - | - |
|  | - | - | - | - | - | Maternity Hospital Data (HES-APC-MAT) | A sub-set of HES-APC for hospital episodes related to maternity and childbirth, including the details of delivery and health information of the baby | 1997-01-01 to  2022-04-25 | Derived from ICD-10 and OPCS-4 | TRE update: Monthly |
| **ONS Census** | Office of National Statistics 2011 Census for Wales (CENW) | A detailed snapshot of the Wales population and its characteristics (including ethnic group and country of birth, health and housing and accommodation) | 2011-03-27 to 2011-03-27 | - | One-off | - | - | - | - | - |
| **Care home** | Care homes index (CARE) | Residential and geographical information data about care homes in Wales | 2018-05-09 to 2020-11-10 | - | Quarterly | - | - | - | - | - |
| **National Institute for Cardiovascular**  **Outcomes Research (NICOR) CVD Audits** | - | - | - | - | - | Percutaneous Coronary Interventions (PCI) | Information of patients going through percutaneous coronary interventions (PCI), including demographic, pre-PCI status, medical history, cardiac anatomy, procedure, and outcome of the PCI | 2017-01-01 to 2022-02-07 | - | TRE update: One to three months |
|  | - | - | - | - | - | Myocardial Ischaemia National Audit Project (MINAP) | Details of care provided to patients with acute coronary syndrome at an emergency department, including patient demographic, admission, examinations, tests, complications, diagnosis, medical history, treatment, medications, interventions, and discharge (e.g., prescription of preventive medications) | 2017-01-01 to  2022-02-12 | - | TRE update: One to three months |
|  | - | - | - | - | - | National Congenital Heart Disease Audit (NCHDA) | Details of service delivery related to infants, children, adolescents, and adults undergoing interventions for paediatric and congenital heart disease, including patient demographics, admission, diagnosis, pre-procedure status, comorbidities, treatment, post-procedure status and complications, outcome, and discharge | 2018-01-01 to  2021-10-28 | European Paediatric Cardiac Code (EPCC) | TRE update: One to three months |
|  | - | - | - | - | - | National Heart Failure Audit (NHFA) | Demographic, diagnosis, signs, symptoms, tests, history of comorbidities, and treatment of patients with an unscheduled hospital admission who are discharged with a primary diagnosis of heart failure | 2018-01-01 to 2022-01-17 | ICD-10 codes for heart failure (I11.0, I25.5, I42.0, I42.9, I50.0, I50.1, I50.9) | TRE update: One to three months |
| **Stroke Audit** | - | - | - | - | - | Sentinel Stroke National Audit Programme clinical dataset (SSNAP) | A prospective minimum dataset for every stroke patient since December 2012 to measure processes of acute care, rehabilitation, care in the community, and outcome measures up to 6 months post admission. The dataset in TRE relates to the patient data submitted to King’s College London under Sentinel Stroke National Audit Programme (SSNAP). | 2014-01-21 to  2022-06-20 | - | TRE update: Monthly |
| **Administrative information** | Welsh Demographic Service Dataset (WDSD) | Register of all individuals registered with a Welsh GP, includes individuals anonymised address and practice history | 1990-01-01 to 2022-08-31 | - | Weekly | - | - | - | - | - |
| **Other** | Referral to Treatment Times Dataset (RTTD) | Information on the total time waited from referral by a GP or other medical practitioner to hospital treatment in the NHS | 2012-01-01 to 2022-09-01 | - | Monthly | - | - | - | - | - |
|  | SAIL Dementia Electronic Cohort (SDEC) | A population-based electronic cohort containing health-related information on people with and without diagnosed dementia (developed by applying coding algorithms to linked routinely-collected datasets) | 2019-03-01 to 2022-02-28 | ICD-10  READ V2 | Quarterly | - | - | - | - | - |
|  | Welsh Ambulance Service Dataset (WASD) | Information about ambulance services (including Emergency Medical Services, and Patient Care Services) | 2013-04-01 to 2022-09-01 | - | Daily | - | - | - | - | - |

See (35) for more information about listed original data sources. ^1^The cut-off start date is set to a date when the count of records with non-duplicated pseudonymised IDs exceeds 10,000. ^2^Pillar 1 Test: swab testing in Public Health England (PHE) labs and NHS hospitals for those with a clinical need, and health and care workers; Pillar 2 Test: swab testing for the wider population, as set out in government guidance; Pillar 3 Test: Serology testing to show if people have antibodies from having had COVID-19. ^3^RRDA: Research Ready Data Asset.
