# Supplementary material for: Harmonising electronic health records for reproducible research: challenges, solutions and recommendations from a UK-wide COVID-19 research collaboration
Source: BMC Med Inform Decis Mak. 2023 Jan 16;23:8. doi: 10.1186/s12911-022-02093-0 (PMC9842203; doi:10.1186/s12911-022-02093-0)
Supplement: Supplementary file 2 — Additional file 2. Microsoft Word (.doc)—Code-lists in SNOMED-CT and Read V2 for COVID-19 diagnosis in primary care data. [file 12911_2022_2093_MOESM2_ESM.docx]

**Additional file 2: Code-lists in SNOMED-CT and Read V2 for COVID-19 diagnosis in primary care data.**

| **SNOMED-CT** | |
| --- | --- |
| Code | Description |
| 1321211000000109 | Coronavirus disease 19 caused by severe acute respiratory syndrome coronavirus 2 presenting complaints simple reference set (foundation metadata concept) |
| 1321341000000103 | Arbitrary concentration of severe acute respiratory syndrome coronavirus 2 immunoglobulin G in serum (observable entity) |
| 1321801000000108 | Arbitrary concentration of severe acute respiratory syndrome coronavirus 2 immunoglobulin A in serum (observable entity) |
| 1321811000000105 | Severe acute respiratory syndrome coronavirus 2 immunoglobulin A qualitative existence in specimen (observable entity) |
| 1240511000000106 | Detection of severe acute respiratory syndrome coronavirus 2 using polymerase chain reaction technique (procedure) |
| 1240571000000101 | Gastroenteritis caused by severe acute respiratory syndrome coronavirus 2 (disorder) |
| 1321241000000105 | Cardiomyopathy caused by severe acute respiratory syndrome coronavirus 2 (disorder) |
| 1321551000000106 | Severe acute respiratory syndrome coronavirus 2 immunoglobulin M detected (finding) |
| 1240581000000104 | Severe acute respiratory syndrome coronavirus 2 ribonucleic acid detected (finding) |
| 1240401000000105 | Antibody to severe acute respiratory syndrome coronavirus 2 (substance) |
| 1240391000000107 | Antigen of severe acute respiratory syndrome coronavirus 2 (substance) |
| 1300681000000102 | Assessment using coronavirus disease 19 severity scale (procedure) |
| 1029481000000103 | Coronavirus nucleic acid detection assay (observable entity |
| 1300631000000101 | Coronavirus disease 19 severity score (observable entity) |
| 1300671000000104 | Coronavirus disease 19 severity scale (assessment scale) |
| 121973000 | Measurement of coronavirus antibody (procedure) |
| 120814005 | Coronavirus antibody (substance) |
| 1240381000000105 | Severe acute respiratory syndrome coronavirus 2 (organism) |
| 1321191000000105 | Coronavirus disease 19 caused by severe acute respiratory syndrome coronavirus 2 procedures simple reference set (foundation metadata concept) |
| 1300721000000109 | Coronavirus disease 19 caused by severe acute respiratory syndrome coronavirus 2 confirmed by laboratory test (situation) |
| 1240541000000107 | Infection of upper respiratory tract caused by severe acute respiratory syndrome coronavirus 2 (disorder) |
| 1322781000000102 | Severe acute respiratory syndrome coronavirus 2 antigen detection result positive (finding) |
| 1240751000000100 | Coronavirus disease 19 caused by severe acute respiratory syndrome coronavirus 2 (disorder) |
| 1240561000000108 | Encephalopathy caused by severe acute respiratory syndrome coronavirus 2 (disorder) |
| 1321541000000108 | Severe acute respiratory syndrome coronavirus 2 immunoglobulin G detected (finding) |
| 1321181000000108 | Coronavirus disease 19 caused by severe acute respiratory syndrome coronavirus 2 record extraction simple reference set (foundation metadata concept) |
| 1321321000000105 | Severe acute respiratory syndrome coronavirus 2 immunoglobulin G qualitative existence in specimen (observable entity) |
| 1321761000000103 | Severe acute respiratory syndrome coronavirus 2 immunoglobulin A detected (finding) |
| 1240411000000107 | Ribonucleic acid of severe acute respiratory syndrome coronavirus 2 (substance) |
| 1240741000000103 | Severe acute respiratory syndrome coronavirus 2 serology (observable entity) |
| 1008541000000105 | Coronavirus ribonucleic acid detection assay (observable entity) |
| 186747009 | Coronavirus infection (disorder) |
| 1321201000000107 | Coronavirus disease 19 caused by severe acute respiratory syndrome coronavirus 2 health issues simple reference set (foundation metadata concept) |
| 1300731000000106 | Coronavirus disease 19 caused by severe acute respiratory syndrome coronavirus 2 confirmed using clinical diagnostic criteria (situation) |
| 1321331000000107 | Arbitrary concentration of severe acute respiratory syndrome coronavirus 2 total immunoglobulin in serum (observable entity) |
| 1321351000000100 | Arbitrary concentration of severe acute respiratory syndrome coronavirus 2 immunoglobulin M in serum (observable entity) |
| 1321301000000101 | Severe acute respiratory syndrome coronavirus 2 ribonucleic acid qualitative existence in specimen (observable entity) |
| 1321311000000104 | Severe acute respiratory syndrome coronavirus 2 immunoglobulin M qualitative existence in specimen (observable entity) |
| 1322871000000109 | Severe acute respiratory syndrome coronavirus 2 antibody detection result positive (finding) |
| 1240521000000100 | Otitis media caused by severe acute respiratory syndrome coronavirus 2 (disorder) |
| 1240531000000103 | Myocarditis caused by severe acute respiratory syndrome coronavirus 2 (disorder) |
| 1240551000000105 | Pneumonia caused by severe acute respiratory syndrome coronavirus 2 (disorder) |
| 1240421000000101 | Serotype severe acute respiratory syndrome coronavirus 2 (qualifier value) |
| **Read V2 (5-character level)** | |
| Code | Description |
| 4J3R1 | 2019-nCoV (novel coronavirus) detected |
| 4J3R. | 2019-nCoV (novel coronavirus) serology |
| A7953 | COVID-19 confirmed using clinical diagnostic criteria |
| A7952 | COVID-19 confirmed by laboratory test |
| A7951 | Disease caused by 2019-nCoV (novel coronavirus) |
